# Comprehensive fecal metagenomic and metabolomic analysis reveals the role of gut microbiota and metabolites in detecting brain metastasis of small cell lung cancer

**DOI:** 10.3389/fmicb.2025.1673983

**Published:** 2025-11-21

**Authors:** Xu Han, Qiang-Guo Sun, Dan Zang, Jun Chen

**Affiliations:** Department of Oncology, The Second Hospital of Dalian Medical University, Dalian, China

**Keywords:** gut microbiota, small cell lung cancer, brain metastasis, metagenomics, metabolomics

## Abstract

**Background:**

Brain metastasis (BM) is a common and highly lethal complication in patients with small cell lung cancer (SCLC). People have paid great attention to exploring the relationship between gut microbiota and the occurrence and development of cancer. This study investigated the relationship between brain metastasis, gut microbiota, and their metabolites in SCLC, providing new insights for the prevention and diagnosis of brain metastasis in SCLC.

**Methods:**

Baseline fecal samples were collected from 45 participants, including 15 patients with BM and 30 patients with no distant metastasis who were newly diagnosed with SCLC. The gut microbiota and metabolite levels were analyzed using metagenomics and untargeted metabolomics, and machine learning models were utilized to identify differences and potential biomarkers.

**Results:**

Gut microbiota composition varied significantly between the two groups. Genus such as *Alistipes* and *Streptococcus* were more abundant in the brain metastasis group, while *Bacteroides* and *Prevotella* predominated in patients without distant spread. Metabolomic profiling identified several metabolites inversely associated with brain metastasis, including leukotriene F4, benzoic acid, velnacrine, piperidine, and an unidentified compound labeled C20916. KEGG pathway analysis linked multiple key physiological processes, such as aminobenzoate degradation, carbapenem biosynthesis, toluene degradation, dioxin degradation, and benzoate degradation, underscoring the complex role of gut microbial metabolites in cancer progression. Furthermore, machine learning models identified key biomarkers, including the genus *Marvinbryantia* and the metabolites benzoic acid, which showed strong discriminatory ability for brain metastasis. After robust validation, the model demonstrated good performance with excellent discriminative power (AUC = 0.80).

**Conclusion:**

Compared to patients without distant metastasis, SCLC patients with BM exhibit distinctive gut microbial and metabolite profiles. These findings suggest that specific gut microbes and their metabolic products may serve as valuable biomarkers for diagnosing and stratifying treatment in brain metastatic SCLC.

## 1 Background

Lung cancer is among the most deadly malignancies in humans, with the highest incidence and mortality rates worldwide (Thai et al., 2021). Based on histopathological features, it is classified into SCLC and non-small cell lung cancer (NSCLC), with SCLC representing approximately 14–15% of all lung cancer cases (Siegel et al., 2021). Originating mainly from neuroendocrine cells, SCLC is considered one of the most difficult cancers to treat, with an overall five-year survival rate below 10% (Wang et al., 2023; Siegel et al., 2024; Kratzer et al., 2024). At diagnosis, about 70% of SCLC patients already have distant metastasis, and common sites of metastasis include the lung, liver, brain, bone, adrenal glands, and lymph nodes (Rudin et al., 2021). Notably, SCLC shows a strong tendency for spread to the central nervous system (CNS), with BM present in roughly 10–15% of patients at first diagnosis (Lukas et al., 2017). Brain-metastatic SCLC progresses quickly and is often mistaken for a primary brain tumor, significantly threatening patient survival. Although multiple treatments have been developed for SCLC, few are effective at preventing distant spread—especially brain metastasis (Siegel et al., 2023). The median survival time for patients with metastatic SCLC is approximately 1 year, and only around 5 months for those with brain involvement (Siegel et al., 2021). Given the poor prognosis linked to BM, there is a critical need to find new biomarkers and treatments to predict and control brain metastasis in SCLC.

The gut microbiota—often called the body’s “second genome”—plays a role in normal physiology and tumor development by influencing immune function, metabolic processes, and neural signaling pathways (Belkaid and Hand, 2014; Lu et al., 2022). During tumor initiation, certain microbial taxa such as *Fusobacterium nucleatum* can cause DNA damage and chronic inflammation, leading to malignant transformation (Riaz et al., 2024; Ivleva and Grivennikov, 2022). Dysbiosis of the gut microbiome is also believed to alter systemic immune responses, which can help cancer cells spread through the bloodstream and settle in the brain (Lu et al., 2021). In brain metastasis, gut microbes and their metabolites, including short-chain fatty acids (SCFAs) can affect blood–brain barrier (BBB) integrity and increase its permeability, thus allowing tumor cells to infiltrate and changing the brain’s immune environment (Braniste et al., 2014; Ahmed et al., 2022). These findings suggest a potential mechanism by which changes in microbes influence the process of brain metastasis in SCLC.

The interaction between gut microbiota, microbial metabolites, and SCLC brain metastasis has thus gained increasing attention. Emerging evidence suggests that specific microbial taxa and their metabolic products may either promote or inhibit tumor cell spread to the brain by altering the tumor microenvironment. These associations imply that the gut microbiome and its metabolic functions may play a crucial role in the progression of BM in SCLC. Identifying microbial biomarkers and metabolic pathways involved in brain colonization offers hope for developing microbiome-based diagnostic tools and new preventive or therapeutic strategies for SCLC BM.

This study uses an integrated metagenomic and untargeted metabolomic analysis approach to explore the relationship between the gut microbiome and brain metastasis of SCLC. By comparing fecal samples from SCLC patients with brain metastasis to those without distant metastasis, we aim to identify the microbiota and metabolic features linked to brain metastasis. The research is expected to offer new insights into the metastasis mechanism of SCLC from the perspective of the gut microbiome and establish a theoretical basis for developing microbiome-targeted therapies.

## 2 Materials and methods

### 2.1 Study population and sample collection

Patients with SCLC confirmed by pathology were recruited at the Second Hospital of Dalian Medical University. Exclusion criteria included: (i) exposure to antibiotics, probiotics, or high-dose corticosteroids within 4 weeks prior to enrollment; (ii) active infectious diseases such as viral hepatitis, HIV, or syphilis; and (iii) a history of autoimmune or gastrointestinal disorders. A total of 45 patients met the eligibility criteria, of whom 15 presented with BM at diagnosis and 30 had no evidence of distant metastasis. Baseline fecal samples were collected before any anti-tumor therapy and immediately stored at −80 °C until analysis.

The study protocol and the informed consent form were approved by the Ethics Review Committee and the Scientific Review Committee of the Second Hospital of Dalian Medical University (Ethics Approval No. 2022-173). Written informed consent was obtained from all participants.

### 2.2 Metagenomic sequencing

Total genomic DNA was extracted from fecal samples using the PowerSoil DNA Isolation kit (Mo Bio Laboratories). DNA quality and quantity were assessed with the Qubit 3.0 Fluorometer (Life Technologies) and 1% agarose gel electrophoresis. Paired-end libraries were prepared with the VAHTS Universal Plus DNA Library Prep Kit (Vazyme Biotech) and sequenced on an Illumina NovaSeq 6000 platform in paired-end mode. Raw sequence reads underwent quality control and adapter trimming with Trimmomatic v0.33, and host-derived reads were removed by aligning to the human reference genome using Bowtie2. Clean reads for each sample were *de novo* assembled into contigs, coding sequences were predicted, and a non-redundant gene catalog was created for downstream taxonomic and functional analysis.

### 2.3 Metabolomic sequencing

Fecal samples were weighed and suspended in an appropriate volume of extraction solvent. Samples underwent bead-beating, sonication, and vacuum centrifugation for solvent removal, followed by reconstitution of the dried extract prior to instrumental analysis. The LC/MS system used for metabolomics analysis consists of the Waters Acquity I-Class PLUS ultra-high-performance liquid chromatography coupled with the Waters Xevo G2-XS QT of high-resolution mass spectrometer. The column employed is the Waters Acquity UPLC HSS T3 column. Raw data collected with MassLynx V4.2 is processed by Progenesis QI software for peak extraction, alignment, and other data processing operations, utilizing the Progenesis QI software with the online METLIN database and public databases for identification. After qualitative and quantitative metabolite profiling, the resulting data were subjected to quality control, compound annotation, differential abundance analysis, and functional enrichment.

### 2.4 Data processing and statistical analysis

Clinical data were analyzed using GraphPad Prism 9.5 and SPSS 25.0. Continuous variables are presented as mean ± standard deviation (SD), while categorical variables are expressed as counts and percentages. For comparisons of normally distributed continuous variables, the Student’s *t*-test was used; non-normally distributed variables were compared with the Mann–Whitney *U* test or the Kruskal–Wallis test, as appropriate. Categorical variables and proportions were compared using the chi-square test or Fisher’s exact test. All statistical tests were two-sided, and a *p*-value <0.05 was considered statistically significant. FDR correction was performed on the *p*-values of species and metabolite level comparisons to ensure their accuracy.

### 2.5 Bioinformatics data analysis

Alpha diversity was assessed using the ACE and Simpson index. Beta diversity was evaluated by calculating Bray–Curtis distance matrices, which were analyzed using principal coordinates analysis (PCoA). The betadisper test was used to validate the reliability of the PERMANOVA results. Differences in species abundance between groups were tested with the Wilcoxon rank-sum test. Differential taxa were further identified through linear discriminant analysis effect size (LEfSe), using a linear discriminant analysis (LDA) score threshold of 3. The top 80 most abundant species were selected for pairwise correlation analysis based on their relative abundances and variances across samples, and a co-occurrence network was constructed.

All detected metabolites were annotated using the KEGG database, the Human Metabolome Database, and the LIPID MAPS structure database. Orthogonal partial least squares discriminant analysis (OPLS-DA) was performed to characterize group-specific metabolic profiles. Differential metabolites were selected based on their variable importance in projection (VIP) scores from the OPLS-DA model combined with univariate criteria (*p*-value or fold change). The OPLS-DA model was configured with a fold-change threshold of 1 and a VIP value of 1. For each candidate metabolite, receiver operating characteristic (ROC) curve analysis was conducted, and the area under the curve (AUC) was calculated. Differential metabolites were further mapped to KEGG pathways for functional analysis.

After normalizing microbial relative abundances, Pearson correlation analysis was performed between differential metabolites and differential taxa. A random forest classifier was built by combining multiple decision trees trained on random subsets of samples and features. Feature selection was performed using *k*-fold cross-validation (StratifiedKFold, *k* = 5 by default). After training in each fold, feature importance was obtained from the corresponding Random Forest model. The top 20 microbial species and metabolites were selected by aggregating importance scores across all folds. The Random Forest Classifier was configured with the following parameters: max_features = “sqrt,” n_estimators = 1,000, and max_depth = 5. The ROC curves were plotted based on the training set.

## 3 Results

### 3.1 Clinical characteristics of study participants

A total of 45 eligible SCLC patients were enrolled, including 30 patients without distant metastasis (N group) and 15 patients with BM (BM group). No significant differences were found between the N and BM groups in demographic variables such as age, sex, smoking and alcohol history, family history of cancer, or body mass index (BMI). These results suggest that baseline characteristics were well matched between the two groups (Table 1).

**Table 1:** Baseline clinical characteristics of study participants.

Characteristic		Non-metastasis (*n* = 30)	Brain metastasis (*n* = 15)	*p*-value
Age (mean ± SD)		63.10 ± 10.94	66.07 ± 6.397	0.339
Gender	Male	21 (70.0%)	11 (73.3%)	0.999
	Female	9 (30.0%)	4 (26.7%)	
Smoking	Absence	12 (40.0%)	3 (20%)	0.315
	Presence	18 (60.0%)	12 (80%)	
Drinking	Absence	18 (60.0%)	11 (73.3%)	0.514
	Presence	12 (40.0%)	4 (26.7%)	
Tumor staging	I–III	7 (23.3%)	0 (0.0%)	0.077
	IV	23 (76.7%)	15 (100.0%)	
Hypertension	Absence	21 (70.0%)	11 (73.3%)	0.999
	Presence	9 (30.0%)	4 (26.7%)	
Diabetes	Absence	27 (90.0%)	13 (86.7%)	0.999
	Presence	3 (10.0%)	2 (13.3%)	
Coronary heart disease	Absence	29 (96.7%)	14 (93.3%)	0.999
	Presence	1 (3.3%)	1 (6.7%)	
Family history of cancer	Absence	23 (76.7%)	8 (53.3%)	0.172
	Presence	7 (23.3%)	7 (46.7%)	
BMI		24.96 ± 4.770	23.29 ± 3.349	0.230

BMI, body mass index.

### 3.2 Metagenomic analysis of the gut microbiome in SCLC patients with/without brain metastasis

**Figure 1:**
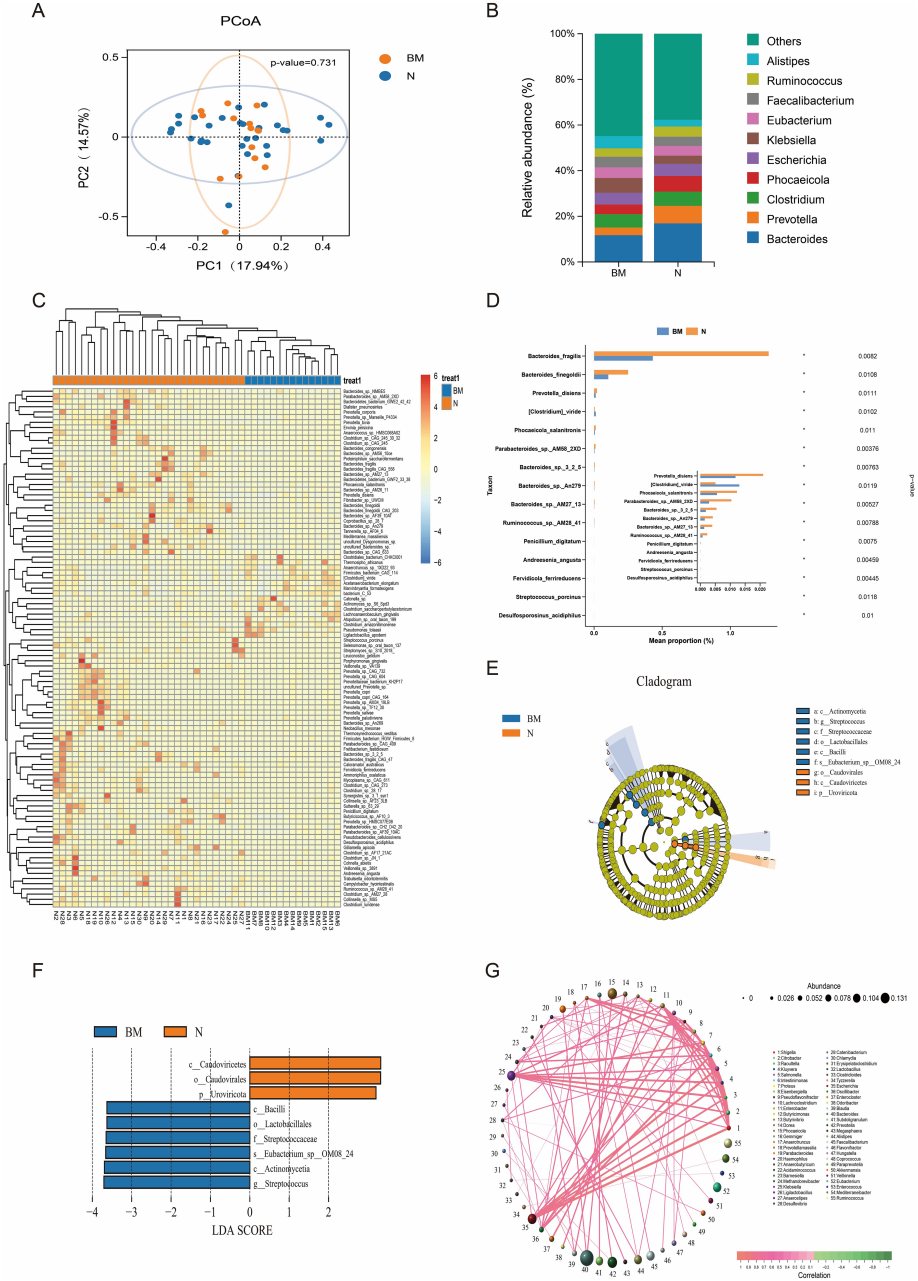
Gut microbiome composition and diversity in SCLC patients with and without brain metastasis. **(A)** Beta diversity analysis based on Bray–Curtis distances. **(B)** Histogram of relative abundances for the top 10 genera. **(C)** Heatmap of differentially abundant species. **(D)** Bar chart showing the abundance of differential species. **(E)** Circular cladogram illustrating taxonomic branching patterns. **(F)** Bar chart of LDA scores for discriminative taxa (LDA ≥3). **(G)** Genus-level co-occurrence network of microbial associations.

Baseline fecal samples from 15 SCLC patients with BM and 30 patients without distant metastasis underwent metagenomic analysis at an average depth of 6 G. In total, 143 phyla, 152 classes, 310 orders, 667 families, 2,278 genera, and 10,739 species were identified. Although the alpha diversity results were not statistically significant, this suggests that alterations in the richness and diversity of the gut microbiota may not significantly impact brain metastasis in SCLC patients (Supplementary Figure 1). In addition to richness and diversity, compositional structure is also an important metric for characterizing the gut microbiota. Therefore, we conducted multiple analyses to explore the compositional differences of the gut microbiota between the two groups. PCoA of Bray–Curtis distances showed clear separation between BM and N samples, indicating significant differences in community structure between the groups (Figure 1A). *Alistipes* are significantly enriched in the gut of patients with brain metastasis. Relatively speaking, *Bacteroides* and *Prevotella* genera were significantly enriched in the intestines of patients without distant metastasis (Figure 1B). At the species levels, many taxa showed differences in abundance between groups (Figure 1C). Notably, species such as *Bacteroides fragilis*, *Bacteroides finegoldii*, and *Prevotella disiens* were significantly reduced in the BM group compared to the N group (Figure 1D). LEfSe analysis at the species level further identified discriminative taxa with an LDA score ≥3: *Caudoviricetes*, *Caudovirales*, and *Uroviricota* were more prevalent in the N group, while *Streptococcus*, *Actinomycetia*, *Eubacterium_sp_OM08_24*, *Streptococcaceae*, *Lactobacillales*, and *Bacilli* were more abundant in the BM group (Figures 1E,F).

To further investigate the significance of gut microbiota dysbiosis in SCLC brain metastasis, we constructed a genus-level correlation network. Significant and complex interrelationships were observed among different taxa, suggesting potential interactions among microbial species. Significant positive correlations were observed among *Escherichia*, *Shigella*, and *Salmonella*, indicating that dysbiosis involving these genera may play a role in disease development. Conversely, canonical probiotics *Lactobacillus* and *Ligilactobacillus*, both involved in SCFAs synthesis, showed a strong negative correlation, highlighting complex metabolic interactions within the gut ecosystem. These interspecies relationships are essential for maintaining microbial ecological balance and host metabolic stability (Figure 1G).

### 3.3 Untargeted metabolomic profiling uncovers metabolic signatures of the gut microbiome in SCLC patients with/without brain metastasis

**Figure 2:**
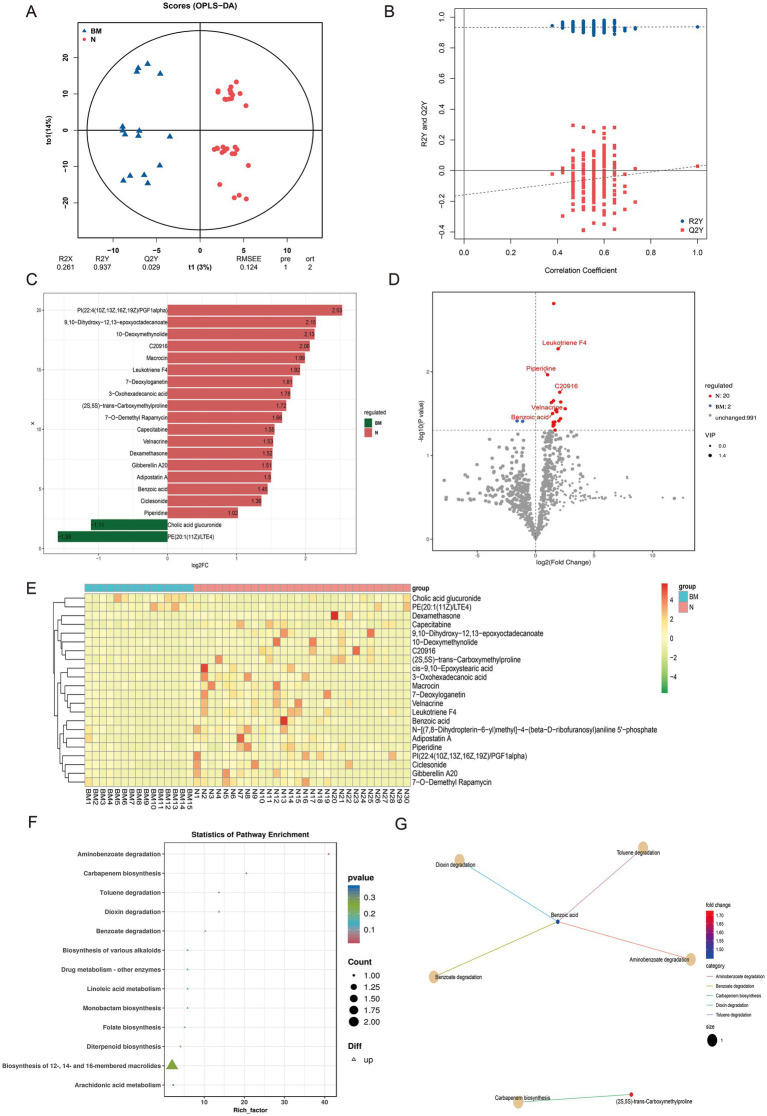
Untargeted metabolomic profiling of fecal samples from SCLC patients with and without brain metastasis. **(A,B)** OPLS-DA score plots showing clear separation of metabolic profiles between the BM and N groups. **(C)** Bar chart of fold changes for the top 20 different metabolites. **(D)** Volcano plot highlighting significantly up- and down-regulated metabolites between BM and N groups. **(E)** Hierarchical clustering heatmap of differential metabolites across all samples. **(F)** KEGG pathway enrichment plot of differential metabolites. **(G)** Network diagram of KEGG pathway enrichment for the differential metabolites.

To further clarify the role of the gut microbiome and its metabolites in SCLC brain metastasis, we performed untargeted metabolomic profiling on 35 baseline fecal samples. Using an LC-QTOF platform, we annotated a total of 1,013 metabolites. OPLS-DA showed a clear separation between brain metastasis (BM) and non-metastatic (N) groups, indicating significant differences in microbial metabolic outputs (Figures 2A,B).

In the BM group, two metabolites were significantly upregulated, including cholic acid glucuronic acid. On the contrary, 20 metabolites including 10-Deoxymethynolide, Macrocin, Leukotriene F4, and benzoic acid were enriched in group N, indicating their potential protective effect on brain transmission (Figures 2C,D). Hierarchical clustering of differential metabolites across all samples identified 12 key compounds; most were specifically enriched in the N group, implying a putative antimetastatic association (Figure 2E).

KEGG pathway enrichment analysis of these differential metabolites revealed their involvement in various biochemical processes, including aminobenzoate degradation, carbapenem biosynthesis, toluene degradation, dioxin degradation, benzoate degradation, and the biosynthesis of different alkaloids (Figure 2F). Notably, benzoic acid showed strong associations with pathways such as aminobenzoate degradation, toluene degradation, dioxin degradation, and benzoate degradation (Figure 2G).

We then assessed the diagnostic potential of five key metabolites—benzoic acid, leukotriene F4, piperidine, C2096, and velnacrine—by comparing their levels between groups and conducting ROC analysis. All five metabolites were significantly higher in the N group (benzoic acid, *p* = 0.032; leukotriene F4, *p* = 0.050; piperidine, *p* = 0.011; C2096, *p* = 0.018; velnacrine, *p* = 0.022) (Supplementary Figure 2A). The ROC curves showed AUC values of these metabolites (Supplementary Figure 2B). Although there were significant differences in the levels of these metabolites between the two groups of patients, ROC analysis results showed that except for benzoic acid, the other metabolites did not perform well in identifying SCLC brain metastasis. At present, our research is only at the level of omics analysis, and more validation may be needed in the future to demonstrate the potential of these metabolites in identifying SCLC brain metastasis. Our research findings, combined with previous studies, suggest that benzoic acid may be a candidate biomarker for predicting brain metastasis in SCLC.

Together, these data suggest that changes in gut microbial metabolism are closely connected to the development of BM in SCLC, and that certain metabolites and pathways might be useful as biomarkers for early detection and treatment.

### 3.4 Integrated multi-omics analysis reveals links between gut microbiota and metabolites in SCLC brain metastasis

**Figure 3:**
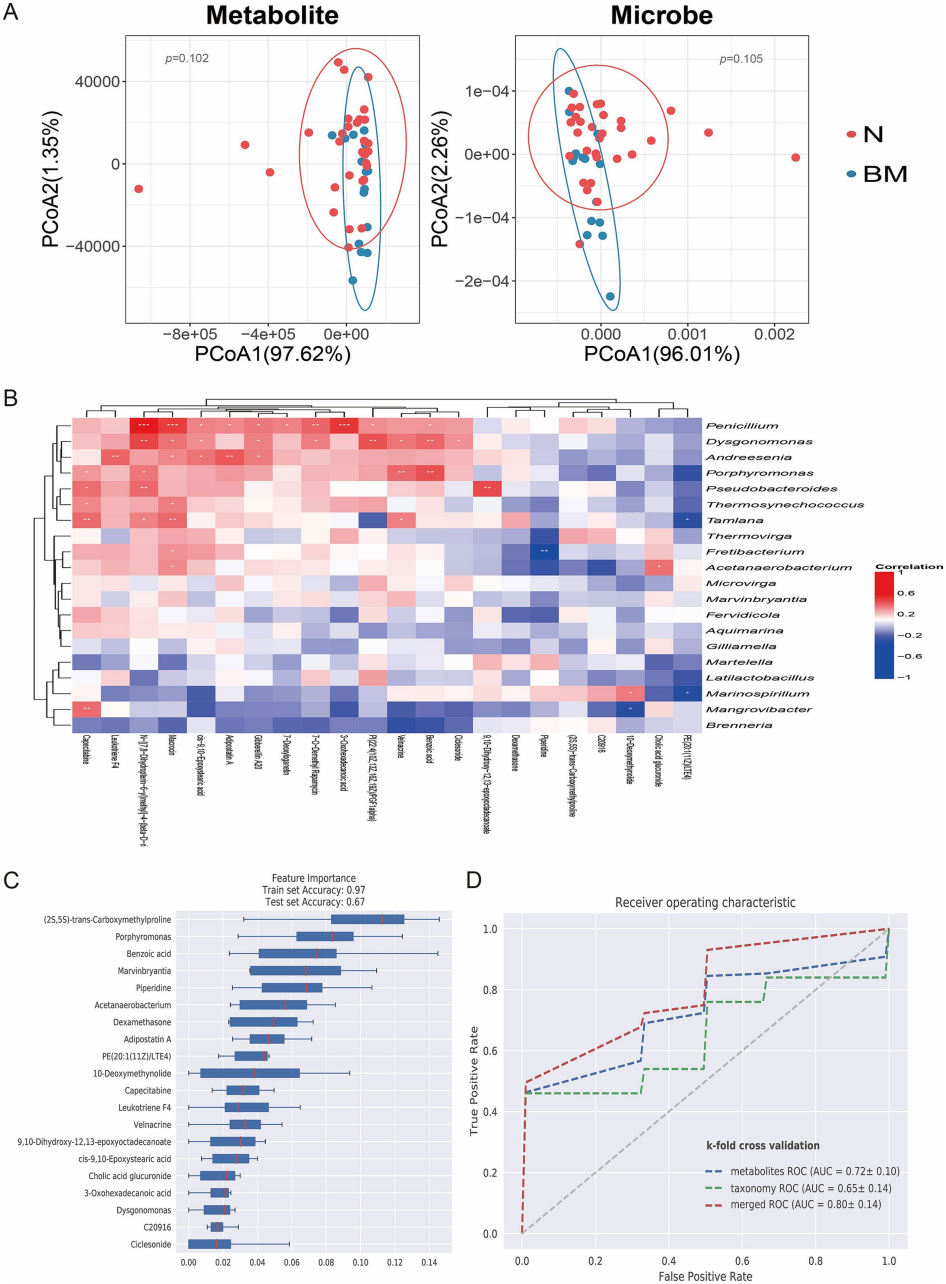
Correlation between gut microbiota and metabolites in SCLC brain metastasis. **(A)** PCoA illustrating clear clustering based on metabolomic and microbiome profiles across groups. **(B)** Heatmap displaying correlations between differential metabolite levels and microbial species abundance. **(C)** Feature importance ranking of key microbial and metabolic biomarkers that differentiate non-metastatic from brain-metastatic SCLC patients using a random forest model. **(D)** ROC curve of random forest model.

To explore the connections between the gut microbiota and its metabolites, we conducted an integrated analysis of metagenomic and metabolomic datasets. Dimensionality reduction and comparative multi-omics analyses uncovered significant differences in microbial community composition, metabolite profiles between the BM and N groups (Figure 3A), indicating that specific biological mechanisms might contribute to SCLC brain metastasis. Correlation analysis showed strong associations between microbial taxa and metabolites (Figure 3B). For instance, *Penicillium* was positively correlated with macrocin, 3-oxohexadecanoic acid, and benzoic acid.

We further used machine learning, specifically a random forest classifier to combine metagenomic and metabolomic features and rank their importance for predicting brain metastasis. Feature importance analysis identified key microbial and metabolic biomarkers, including the bacterial genus *Marvinbryantia* and metabolites such as benzoic acid, which played crucial roles in distinguishing BM from N samples (Figure 3C). We further compared the ROC curves of metagenomics, metabolomics and merged analysis. The results showed that the merged analysis could more accurately distinguish the brain metastasis group from the non-metastasis group, suggesting that the multi-omics merged analysis model has a better predictive effect on brain metastasis (Figure 3D). These findings offer deeper insight into the molecular traits linked to brain dissemination and highlight the significance of integrated multi-omics approaches for biomarker discovery in SCLC brain metastasis.

## 4 Discussion

Brain metastasis is a serious and life-threatening complication of SCLC. About 15% of SCLC patients have BM at diagnosis, and their median survival is significantly shorter than those without brain involvement (Li et al., 2021). Nearly 60% of SCLC patients develop BM within 3 years of their initial diagnosis, and overall survival after brain metastasis is less than 5 months (Aupérin et al., 1999; Chen et al., 2018). It is well known that the gut microbiota plays a key role in regulating host metabolism and supporting immune balance; dysbiosis can cause inflammation and alter the tumor immune microenvironment, helping metastatic spread (Li et al., 2025). A growing body of evidence suggests that the gut microbiota can regulate the structure and function of the blood–brain barrier through various mechanisms, thereby potentially influencing the colonization and metastasis of tumor cells in the brain. Gut dysbiosis can lead to the entry of endotoxins (such as lipopolysaccharide) into the circulation, triggering a systemic inflammatory response and activating peripheral pro-inflammatory cytokines. These inflammatory mediators can downregulate endothelial tight junction proteins and increase BBB permeability (Sharon et al., 2016). Furthermore, the level of oxidative stress regulated by the gut microbiota is also crucial for BBB homeostasis, as the accumulation of reactive oxygen species and insufficient antioxidant defenses are closely associated with endothelial barrier damage (Fung et al., 2017). Microbial metabolites, such as SCFAs, can upregulate the expression of tight junction proteins (e.g., occludin and claudin-5), thereby maintaining BBB integrity. In contrast, gut microbiota imbalance leads to reduced SCFAs levels, increased BBB permeability, and enhanced trans-barrier capability for exogenous molecules and circulating tumor cells (Braniste et al., 2014). Tryptophan metabolites regulate endothelial cell function and glial cell activation through the aryl hydrocarbon receptor signaling pathway. Abnormal tryptophan metabolism can cause endothelial inflammation and barrier disruption, creating favorable conditions for tumor cells to enter the central nervous system (Rothhammer and Quintana, 2019). In the context of tumor metastasis, these mechanisms may collectively act to make the BBB more susceptible to penetration by circulating tumor cells in specific microenvironments, thereby promoting the occurrence of brain metastasis. Additionally, the makeup of the gut microbiome and its metabolites is increasingly recognized as both a diagnostic and treatment tool in cancer (Coker et al., 2022; Lee et al., 2022). Although growing evidence links gut microbes to the development and progression of tumors, research specifically focused on microbiome changes in SCLC patients with BM remains limited, and potential microbial biomarkers for brain metastasis have not yet been fully investigated.

Therefore, we conducted a study on 45 subjects, including 30 SCLC patients without distant metastasis and 15 SCLC patients with BM, to examine the role of gut microbiota and metabolites in the progression of SCLC BM. These findings may provide new insights into the involvement of gut microbiota in the diagnosis and treatment of SCLC brain metastasis.

We observed clear differences in gut community structure between BM and N groups, consistent with prior reports indicating that microbial diversity correlates with lung cancer progression and metastatic potential (Liu et al., 2019). Such changes in microbial ecology may create a pro-metastatic niche by influencing host immune responses and metabolic pathways.

At the genus level, *Alistipes* was significantly more abundant in BM patients, while *Bacteroides* and *Prevotella* dominated in the N group. *Alistipes* has been linked to colorectal tumor formation and was recently shown to promote lung cancer growth and reduce immunotherapy effectiveness (Yang et al., 2022; Rahal et al., 2024). Interestingly, we found *Bacteroides fragilis* and *Bacteroides finegoldii* enriched in non-metastatic patients, although *enterotoxigenic Bacteroides fragilis* colonization has been associated with epithelial cell growth and breast cancer metastasis in other contexts (Parida et al., 2021). These different studies suggest that this subspecies may play a more complex and diverse role in cancer metastasis, with different effects under different conditions and environments. Further research is needed to explore the mechanism of action of this subspecies. We also observed enrichment of pathobionts like *Streptococcus* in BM patients, supporting the idea that dysbiosis can create an immunosuppressive microenvironment and drive epithelial–mesenchymal transition, thus promoting metastatic invasion (Lu et al., 2025; Oehmcke-Hecht et al., 2020).

Beyond compositional shifts, gut microbes influence systemic functions through metabolite production. Key microbial metabolites, such as SCFAs, bile acids, and tryptophan derivatives, have been shown to affect endothelial permeability, immune cell movement, and cancer-related signaling pathways, all of which impact cancer cell metastatic colonization (Chen et al., 2020; Li et al., 2024; Gou et al., 2024). Our untargeted metabolomics identified significant differences of multiple metabolites, including leukotriene F4, benzoic acid, velnacrine, piperidine, and C20916, which were negatively associated with BM. KEGG enrichment analysis pointed to pathways such as aminobenzoate degradation, carbapenem biosynthesis, toluene degradation, dioxin breakdown, and benzoate degradation—highlighting potential microbiome–host interaction pathways in SCLC brain metastasis. The enrichment of aromatic hydrocarbon metabolic pathways such as benzoate degradation, dioxin degradation, and toluene degradation suggests alterations in the gut microbiota’s processing of exogenous or endogenous aromatic compounds. Benzoate often originates from the degradation of dietary polyphenols or from the food additive sodium benzoate. Absorbed benzoate is almost entirely excreted in the form of hippurate (Yadav et al., 2021), while the gut microbiota possesses genes related to benzoate degradation, enabling it to further metabolize benzoate into intermediate products such as catechol. Products of the catechol pathway possess antioxidant and immunomodulatory effects, and changes in their metabolic levels may reflect an increase in inflammatory and oxidative stress states. Dioxins are a class of persistent organic pollutants that can act as potent ligands for the aryl hydrocarbon receptor, regulating barrier immunity and inflammatory responses in the gut and brain. Long-term exposure to dioxin-like compounds can lead to chronic intestinal inflammation, oxidative damage, and disruption of the blood–brain barrier, potentially creating favorable conditions for brain metastasis of tumor cells (Coretti et al., 2024). Toluene, also an aromatic hydrocarbon, has strong neurotoxicity and can easily cross the blood–brain barrier, inducing oxidative stress and inflammation in the nervous system. The enrichment of these environmental or endogenous aromatic hydrocarbon degradation pathways reflects an enhanced degradation activity of the gut microbiota towards toluene and its metabolites (such as benzoate). It has been reported in the literature that environmental pollutants can alter the gut-brain axis by disrupting the gut microbiota, leading to changes in central nervous system function (Singh et al., 2022). Therefore, the enrichment of benzoate, dioxin, toluene degradation pathways in this study suggests that the gut microbiota’s degradation of substances that adversely affect blood–brain barrier permeability, along with its active regulation of barrier immunity and inflammatory responses in the gut and brain, may help prevent the occurrence and development of lung cancer brain metastasis. Notably, benzoic acid, a major intermediate involved in multiple metabolic pathways, has been reported to inhibit histone deacetylases and reduce tumor cell growth, implying a protective effect against brain metastasis (Anantharaju et al., 2017). At present, the FDA has approved more than 2,000 anti-cancer drugs containing piperidine, and the development of small-molecule drugs containing piperidine as anti-cancer drugs is also actively underway, emphasizing the good application of piperidine in resisting tumor occurrence and development (Goel et al., 2018).

Our integrated multi-omics analysis offers a comprehensive view of the complex interaction between gut microbial taxa and their metabolic outputs in SCLC brain metastasis. Machine learning identified *Marvinbryantia* and metabolites such as benzoic acid as top predictors of brain metastasis, emphasizing the potential of microbiome-based biomarkers for early detection and monitoring. *Marvinbryantia*, as a key butyrate producer in the Firmicutes phylum, may synthesize butyric acid through the acetyl CoA pathway, thereby regulating intestinal barrier function and systemic immune response (He et al., 2025). In the context of lung cancer brain metastasis, a decrease in its abundance may lead to a decrease in butyric acid levels, weakening the anti-inflammatory and immune regulatory effects mediated by butyric acid. On the one hand, butyric acid promotes regulatory T cell differentiation by inhibiting histone deacetylase (HDAC) and suppressing excessive inflammation. On the other hand, it may enhance dendritic cell activation and infiltration of CD8^+^ T cells into the tumor microenvironment, similar to the synergistic mechanism of *Akkermansia* in immunotherapy (Sivan et al., 2015; Routy et al., 2018). Although there is limited direct experimental data on *Marvinbryantia* in the current evidence chain, its functions in short chain fatty acid metabolism, toxic substance clearance, and immune microenvironment regulation support the rationality of its involvement in lung cancer brain metastasis through the “gut immune brain axis.” This provides a theoretical basis for the development of probiotic interventions or microbial marker panels targeting this bacterium.

This study also has limitations. As a single-center investigation with a modest sample size, our findings need validation in larger, multi-institutional cohorts to confirm their generalizability. Given the relatively low clinical incidence of SCLC, the sample size included in this study is small, which limits statistical power and increases the risk of overfitting in machine learning models. Additional cohorts are required to further verify performance stability. However, current research on SCLC brain metastasis and gut microbiota remains scarce, making it regrettable that external data cannot be used to validate the experimental findings. We propose prioritizing the generation of hypotheses from this exploratory cohort, with the hope that future studies will validate the feasibility and accuracy of machine learning models predicting SCLC brain metastasis based on gut microbiota and metabolites. Additionally, although our correlative analyses suggest links between dysbiosis, specific metabolites, and brain metastasis. Mechanistic studies, including external validation cohorts and functional experiments are necessary to establish causality and clarify underlying pathways.

In summary, our data reveal distinct gut microbial and metabolic signatures in SCLC patients with BM and support the idea of using microbiome-derived biomarkers and interventions to predict and reduce brain metastasis in this aggressive disease.

## 5 Conclusion

**Figure 4:**
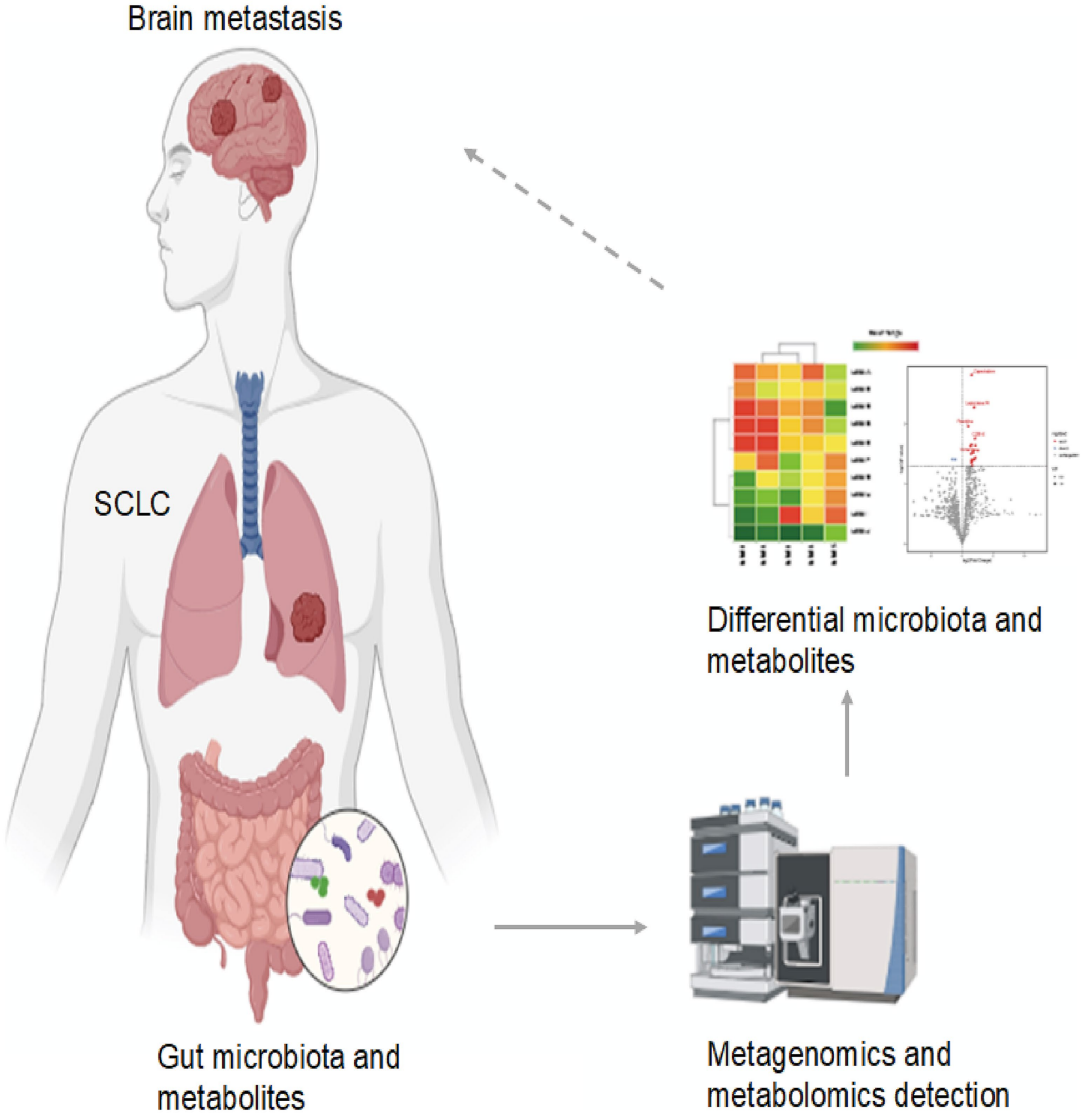
The role of gut microbiota and metabolites as potential biomarkers in SCLC brain metastasis.

This study emphasizes the crucial role of the gut microbiota and its metabolic products in the development of BM in SCLC patients. We found distinct microbial and metabolite signatures in patients with cerebral dissemination compared to those without distant metastasis, providing evidence that dysbiosis may aid SCLC spread to the brain. Our results also suggest that specific gut microbes and their metabolites show promise as auxiliary biomarkers for monitoring brain metastasis in SCLC, pending prospective validation. These microbial biomarker panels offer a promising approach for early, minimally invasive diagnosis in affected patients (Figure 4). Lastly, our findings highlight the need for larger, comprehensive studies to better understand the complex relationship between the gut microbiome, its metabolic outputs, and SCLC brain metastasis.

## Data Availability

The raw sequencing data and processed metabolomics data presented in the study is openly available in Science Data Bank, DOI: 10.57760/scientificdb.28513.
