# Supplementary material for: Comprehensive fecal metagenomic and metabolomic analysis reveals the role of gut microbiota and metabolites in detecting brain metastasis of small cell lung cancer
Source: Front Microbiol. 2025 Nov 21;16:1673983. doi: 10.3389/fmicb.2025.1673983 (PMC12679941; doi:10.3389/fmicb.2025.1673983)

Supplementary Figure

Supplementary Figure

Supplement Figure 1. Alpha diversity analysis of SCLC Patients With and Without Brain Metastases. (A) ACE index between BM and N groups. (B) Simpson index between BM and N groups.

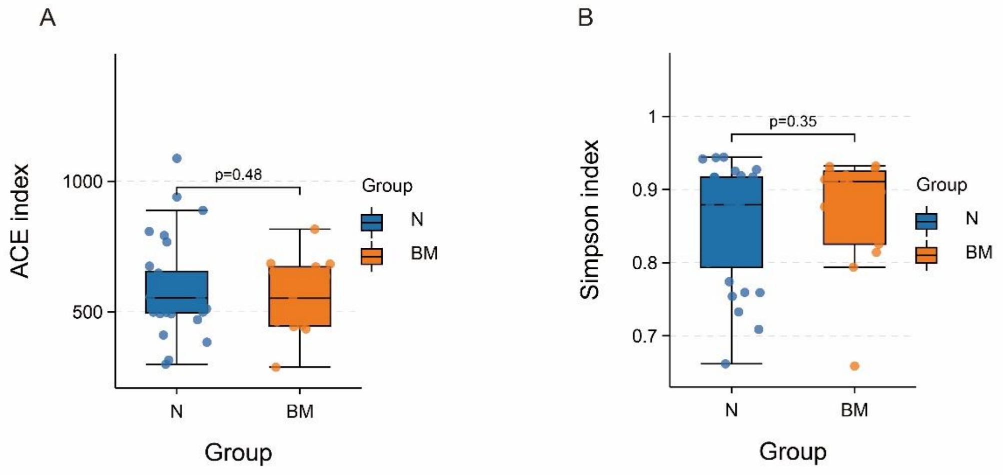

Supplement Figure 2. Diagnostic Potential of Differential Metabolites in SCLC Patients With and Without Brain Metastases. (A) Violin plots displaying the distribution of key differential metabolites between BM and N groups. (B) ROC curves and associated box plots demonstrating the diagnostic performance and group-wise abundance of selected metabolites.

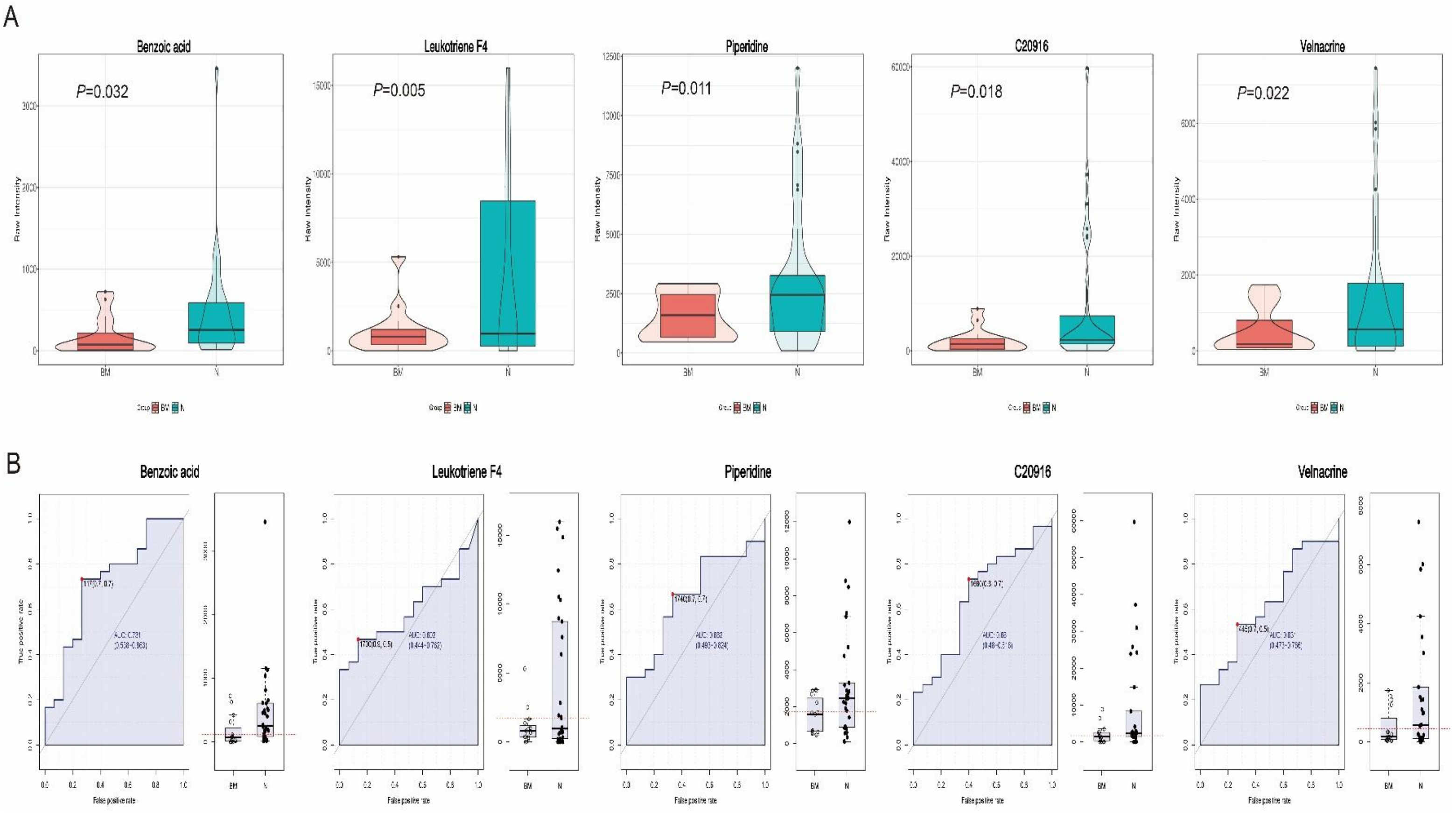

Supplement: Supplementary file 1 [file Presentation_1.pdf]
